# NR1D1 Deletion Induces Rupture-Prone Vulnerable Plaques by Regulating Macrophage Pyroptosis via the NF-*κ*B/NLRP3 Inflammasome Pathway

**DOI:** 10.1155/2021/5217572

**Published:** 2021-12-16

**Authors:** Zhinan Wu, Fei Liao, Guqing Luo, Yuxuan Qian, Xinjie He, Wenyi Xu, Song Ding, Jun Pu

**Affiliations:** Department of Cardiology, Renji Hospital, Shanghai Jiao Tong University School of Medicine, Shanghai, China

## Abstract

Vulnerable plaque rupture is the main trigger of most acute cardiovascular events. But the underlying mechanisms responsible for the transition from stable to vulnerable plaque remain largely unknown. Nuclear receptor subfamily 1 group D member 1 (NR1D1), also known as REV-ERB *α*, is a nuclear receptor that has shown the protective role in cardiovascular system. However, the effect of NR1D1 on vulnerable plaque rupture and its underlying mechanisms are still unclear. By generating the rupture-prone vulnerable plaque model in hypercholesterolemic ApoE^−/−^ mice and NR1D1^−/−^ApoE^−/−^ mice, we demonstrated that NR1D1 deficiency significantly augmented plaque vulnerability/rupture, with higher incidence of intraplaque hemorrhage (78.26% vs. 47.82%, *P* = 0.0325) and spontaneous plaque rupture with intraluminal thrombus formation (65.21% vs. 39.13%, *P* = 0.1392). *In vivo* experiments indicated that NR1D1 exerted a protective role in the vasculature. Mechanically, NR1D1 deficiency aggravates macrophage infiltration, inflammation, and oxidative stress. Compared with the ApoE^−/−^ mice, NR1D1^−/−^ApoE^−/−^ mice exhibited a significantly higher expression level of pyroptosis-related genes in macrophages within the plaque. Further investigation based on mice bone marrow-derived macrophages (BMDMs) confirmed that NR1D1 exerted a protective effect by inhibiting macrophage pyroptosis in a NLRP3-inflammasome-dependent manner. Besides, pharmacological activation of NR1D1 by SR9009, a specific NR1D1 agonist, prevented plaque vulnerability/rupture. In general, our findings provide further evidences that NR1D1 plays a protective role in the vasculature, regulates inflammation and oxidative stress, and stabilizes rupture-prone vulnerable plaques.

## 1. Introduction

Atherosclerosis, characterized by the formation of plaques containing lipids, cells, debris, and scar tissue in the arterial walls, causes much morbidity and mortality worldwide [1, 2]. While atherosclerosis is a chronic inflammation induced by cholesterol, inflammation could alter vascular function and contribute to the atherosclerotic plaque progression in a lipid-independent manner [3]. Rupture at the site of a vulnerable atherosclerotic plaque is the most frequent cause of acute coronary syndrome, but mechanisms responsible for plaque vulnerability and rupture remain largely unknown [4]. It is well known that inflammation contributes to the vulnerability of plaques which are the substrate of acute coronary syndrome [5]. Recent studies demonstrated that macrophage pyroptosis initiated by inflammation conversely augmented the inflammatory response in the vasculature and contributed to the rupture of vulnerable plaques [6, 7].

Pyroptosis is an inflammatory form of programmed cell death characterized by the formation of gasdermin- (GSDM-) based pores on the plasma membrane, which is triggered by the binding of damage/danger-associated molecular patterns (DAMPs) or pathogen-associated molecular patterns (PAMPs) by inflammasomes and pattern recognition receptors (PRR) [8, 9]. Canonical pathways initiate from the activation of the inflammasome complex (NLRP3, NLRP1, NAIP-NLRC4, AIM2, and Pyrin), which provides a platform for the maturation of caspase-1. Caspase-1 leads to the activation of interleukin- (IL-) 1*β* and IL-18, as well as gasdermin D (GSDMD). Activated inflammasome induces formation of cytoplasmic membrane pores, swelling, and rapid cell rupture and thus releases proinflammatory factors and cell contents. Of interest, mounting evidences suggested that pyroptosis of macrophages within atherosclerotic plaques may contribute to plaque vulnerability/rupture [7, 10]. Hence, a more specific understanding of mechanisms underlying macrophage pyroptosis in vulnerable plaques is urgent and may provide a novel therapeutic target for the rupture-prone vulnerable plaques and consequent cardiovascular events.

Nuclear receptor subfamily 1 group D member 1 (NR1D1), also known as REV-ERB *α*, is a member of the nuclear receptor superfamily, functioning as a transcriptional repressor [11]. NR1D1 and other circadian clock proteins (RORs, BMAL, CLOCK, PER, and CRY) are involved in many physiological processes, including regulation of metabolism, development, immunity, and circadian rhythm [12]. Interestingly, emerging evidence suggests that NR1D1 exerts a powerful anti-inflammatory and antioxidative stress effect [13]. Recent studies identified its protective role in various cardiovascular diseases including myocardial infarction and cardiac hypertrophy [14–16].

By using loss-of-function approach (global NR1D1^−/−^ mice) and pharmacological activation of NR1D1 by SR9009, we aimed at determining the effect and molecular mechanisms of NR1D1 on atherosclerotic plaque vulnerability/rupture and the potential involvement of NR1D1 in mediating the vasoprotective effects.

## 2. Materials and Methods

### 2.1. Animal Models of Rupture-Prone Vulnerable Plaques

Animal experiments were referred to the National Institutes of Health Guidelines on the Use of Laboratory Animals and followed the institutional guidelines outlined by the Animal Ethics Committee of Shanghai Jiao Tong University.

NR1D1-deficient (NR1D1^−/−^) mice on a C57BL/6 background were kindly given by Dr. Ueli Schibler (University of Geneva, Geneva, Switzerland) [17]. The NR1D1^−/−^ mice were bred with ApoE^−/−^ mice to establish NR1D1^−/−^ mice under an ApoE^−/−^ background. A total of 23 NR1D1^−/−^ApoE^−/−^ male mice and 69 ApoE^−/−^ male mice at 8 weeks of age were maintained for subsequent study.

For animal models of rupture-prone vulnerable plaques, we used a previously published protocol [18]. In brief, eight-week-old male mice were allowed to acclimate for a week prior to the study and were fed a high-fat diet (1.25% cholesterol and 21% fat; 42% kcal as fat) during the experiment. The rupture-prone vulnerable carotid plaques were induced by partly ligating the left renal artery and the left internal and the external carotid arteries by using perivascular collars. Plaque rupture was defined by an area of fibrous cap disruption. Intraplaque hemorrhage is defined as the deposition of blood products inside the plaque and is not necessarily associated with atherosclerotic plaque rupture. All the mice underwent general anesthesia using pentobarbital sodium at a dose of 100 mg/kg and were maintained on a heating pad. To study the effect of SR9009 on plaque vulnerability/rupture, mice after surgery were randomly divided into the vehicle group (phosphate buffer saline (PBS)) and the SR9009-treated group (100 mg/kg/d, MCE, cat.no. HY-16989, dissolved in PBS and given by intraperitoneal injection once a day) [19, 20].

### 2.2. Microultrasound Imaging

At baseline and postoperative weeks 4 and 8, microultrasound images were obtained with a 55 MHz transducer via the Vevo 770 system (VisualSonics) under isoflurane (2%) inhalation anesthesia. Prior to the ultrasound examination, the hair around the necks of the mice was shaved for better image quality. Standard techniques were used to obtain B-mode measurements of the length of the atherosclerotic lesion in the left common carotid artery and extreme values of intimal-medial thickness (IMT). Additionally, the maximal flow velocity (*V*max) proximal to the stenosis was obtained in a pulse-wave Doppler mode. To measure the degree of eccentric plaque distribution, the eccentric index (EI) was calculated from the extreme values of IMT by the formula (maximal IMT − minimal IMT)/maximal IMT.

### 2.3. Tissue Collection and Analysis

At postoperative week 8, mice were sacrificed by deep anesthesia for tissue collection after measuring blood pressure and body weight. Plasma was obtained from the blood samples of mice by centrifugation at 1000 g for 15 minutes at 4°C and was stored at −80°C. After perfusion, the specimens of the carotid arteries were obtained and kept in paraffin or embedded in OCT compound (Sakura Finetek USA Inc., Torrance, CA, United States).

The levels of the cytokines IL-1*β*, IL-4, IL-10, tumor necrosis factor-*α* (TNF-*α*), superoxide dismutase (SOD), malondialdehyde (MDA), glutathione (GSH), and catalase (CAT) were measured using corresponding ELISA kits (Abcam) following the manufacturers' protocol. Triglyceride (TG) and total cholesterol were measured according to the manufacturer's protocols using commercial kits (Jiancheng Bioengineering Institute Nanjing, China).

### 2.4. Histopathology and Immunofluorescence/Immunohistochemistry

The carotid arteries specimens were embedded in the OCT compound and serially sectioned (5 *μ*m). Hematoxylin and eosin (H&E), Masson's trichrome, and Sirius red staining were performed according to the manufacturers' protocols (Sigma). To evaluate the lipid content in the vasculature, sections were stained with oil red O, using Mayer's hematoxylin (Sigma) as the counter stain.

For immunofluorescence analysis, serial paraffin sections were incubated with different primary antibodies overnight at 4°C, followed by the corresponding fluorochrome-conjugated antibodies for detection. The following primary antibodies were used: rat monoclonal antibody against CD68 (Abcam, ab53444), rabbit monoclonal antibody against NLRP3 (Abcam, ab270449), rabbit monoclonal antibody against IL-1*β* (Abcam, ab234437), rabbit polyclonal antibody against caspase-1 (ABclonal, A16792), and rabbit monoclonal antibody against NR1D1 (Abcam, ab174309). After they reacted with the corresponding Alexa Fluor 488 or 594-conjugated secondary antibodies (Invitrogen), DAPI was added to visualize nuclei. Fluorescent images were acquired with a laser scanning confocal microscope (Leica TCS SP8, Leica Microsystems). All the images and staining intensities were acquired and measured with Image-Pro Plus 6.0 (Media Cybernetics Inc.).

### 2.5. Cell Culture and Treatment

The human endothelial cell line EA.hy 926, murine aortic vascular smooth muscle cell line Movas and murine macrophage cell line Raw 264.7 were purchased from the American Type Culture Collection (ATCC). The EA.hy 926, Movas, and Raw 264.7 were cultured in a 5% CO_2_ incubator. All cells were cultured in Dulbecco's minimum essential medium (DMEM) supplemented with 10% fetal bovine serum (FBS) and 1% penicillin-streptomycin (P/S). To explore the change of NR1D1 expression *in vitro*, EA.hy 926, Movas, and Raw 264.7 were treated with ox-LDL (10 or 20 or 50 *μ*g/mL) for 24 hours.

### 2.6. BMDM Culture and Treatment

Eight- to ten-week-old C57/B6 mice were euthanized according to the animal protocol approved by the Animal Ethics Committee of Shanghai Jiao Tong University. Femurs were flushed with PBS, and mononuclear phagocyte progenitor cells from the bone marrow were collected in the medium. The medium was centrifuged at 1000 rpm for 5 minutes. After removing the supernatant, the precipitate was incubated with red cell lysate at 4°C for 5 minutes and centrifuged again. The cells were plated at the density of 2 × 10^6^/mL in six-well culture plates and cultured in DMEM supplemented with 10% FBS and 1% P/S and 20 ng/mL M-CSF (PeproTech). Culture media were replaced on day 3 and day 6. On day 7, BMDMs were administrated with ox-LDL (50 *μ*g/mL) and SR9009 (10 *μ*mol/L) [20–22].

### 2.7. Western Blotting

Following standard protocols, proteins were extracted from BMDMs and resolved by 10%–12% SDS-PAGE. Protein extracts were subsequently electrotransferred onto polyvinylidene fluoride membranes followed by overnight incubation at 4°C with corresponding primary antibodies. The following primary antibodies were used: rabbit monoclonal antibody against NR1D1 (Abcam, ab174309), rabbit monoclonal antibody against NLRP3 (Abcam, ab270449), rabbit monoclonal antibody against IL-1*β* (Abcam, ab234437), rabbit polyclonal antibody against caspase-1 (ABclonal, A16792), rabbit monoclonal antibody against GSDMD (Abcam, ab209845), rabbit polyclonal antibody against phospho-NF-*κ*B P65-S536 (Abcam, ab76302), rabbit monoclonal antibody against NF-*κ*B p65 (Abcam, ab32536), rabbit monoclonal antibody against GAPDH (ProteinTech, 60004-1-Ig), and rabbit monoclonal antibody against *α*-tubulin (ProteinTech, 66031-1-Ig). After incubating with the corresponding secondary antibodies (Jackson, 1 : 10000) for 1 hour at room temperature, the immunoblot bands were detected via enhanced chemiluminescence (Millipore, Billerica, MA). A prestained color protein ladder (Thermo) was utilized to calculate the molecular weights of proteins. The membranes were exposed to an autoradiography film with incubation in an enhanced chemiluminescence imaging system (Millipore, Billerica, MA). The signal intensities were analyzed by Gel-Pro Analyzer 4.0 software (Media Cybernetics, Silver Spring, MD).

### 2.8. Real-Time Quantitative PCR

Total RNA was extracted from carotid arteries or BMDMs using TRIzol Reagent (Invitrogen). cDNA was synthesized using RT SuperMix for qPCR (Vazyme) and then amplified by SYBR qPCR master mix (Vazyme) in the LightCycler® 480 Real-Time PCR System (Roche). The primer sequences utilized for real-time PCR are presented in Table S1. Results from LightCycler 480 analysis software are presented as *Ct* values, normalized against GAPDH, and shown as 2^−ΔΔ*Ct*^.

### 2.9. Oxidative Stress Detection In Vivo and In Vitro

Reactive oxygen species (ROS) generation in the vasculature was measured by the dihydroethidium (DHE) staining. Briefly, serial frozen sections of carotid arteries (5 *μ*m) were incubated with DHE (5 *μ*mol/L) in a light-protected humidified chamber at room temperature for 60 min. After incubation, DAPI was added to visualize the nuclei. Fluorescent images were acquired with a laser scanning confocal microscope (Leica TCS SP8, Leica Microsystems). All the images and staining intensities were acquired and measured with Image-Pro Plus 6.0 (Media Cybernetics Inc.).

For detecting reactive oxygen species (ROS) generation in BMDMs, 12-well plates precoated with Matrigel solution (BD) were stored at 4°C overnight. BMDMs were seeded on the precoated plates and incubated at 37°C and 5% CO_2_. After administration of ox-LDL and SR9009, BMDMs were fixed with 4% paraformaldehyde. After being permeabilized in Triton X-100 and blocked in PBS containing 10% BSA, BMDMs were successively stained with 2′,7′-dichlorodihydrofluorescein diacetate (DCFH-DA) (10 *μ*M, Sigma) for 30 minutes at room temperature. After incubation, DAPI was added to visualize the nuclei. Cells were examined with a laser scanning confocal microscope (Leica TCS SP8, Leica Microsystems). The percentage of DCFH-DA-positive cells was analyzed with Image-Pro Plus 6.0 (Media Cybernetics Inc.).

### 2.10. Terminal Deoxynucleotidyl Transferase dUTP Nick End Labelling (TUNEL) Assay

DNA damage of macrophages in carotid plaque lesions was detected by the In Situ Cell Death Detection Kit (Roche, Mannheim, Germany). Serially sectioned paraffin carotid artery specimens (5 *μ*m) were incubated with anti-CD68 primary antibodies (Abcam, ab53444). Sections were then incubated with TUNEL reaction mixture according to the manufacturer's protocol. After incubation, DAPI was added to visualize the nuclei.

To detect DNA damage of BMDMs after administration of ox-LDL and SR9009, BMDMs were fixed with 4% paraformaldehyde. After being permeabilized in Triton X-100 and blocked in PBS containing 10% BSA, BMDMs were successively stained with TUNEL reaction mixture according to the manufacturer's protocol. After incubation, DAPI was added to visualize the nuclei. Images were acquired with a laser-scanning confocal microscope (Leica TCS SP8, Leica Microsystems). All the images and staining intensities were acquired and measured with Image-Pro Plus 6.0 (Media Cybernetics Inc.).

### 2.11. Statistical Analysis

All experiments were blinded and repeated at least three times independently, and similar results were obtained. Continuous variables are performed using IBM-SPSS v.24.0 statistical software (IBM) as the mean ± SEM and analyzed with one-sided *t* test for two independent samples or one-way ANOVA followed by post hoc Tukey's test for sets of data. Categorical variables were compared using chi-square analysis. *P* values were calculated with a 95% confidence level.

## 3. Results

### 3.1. NR1D1 Expression Is downregulated during Vulnerable Plaque Formation

**Figure 1:**
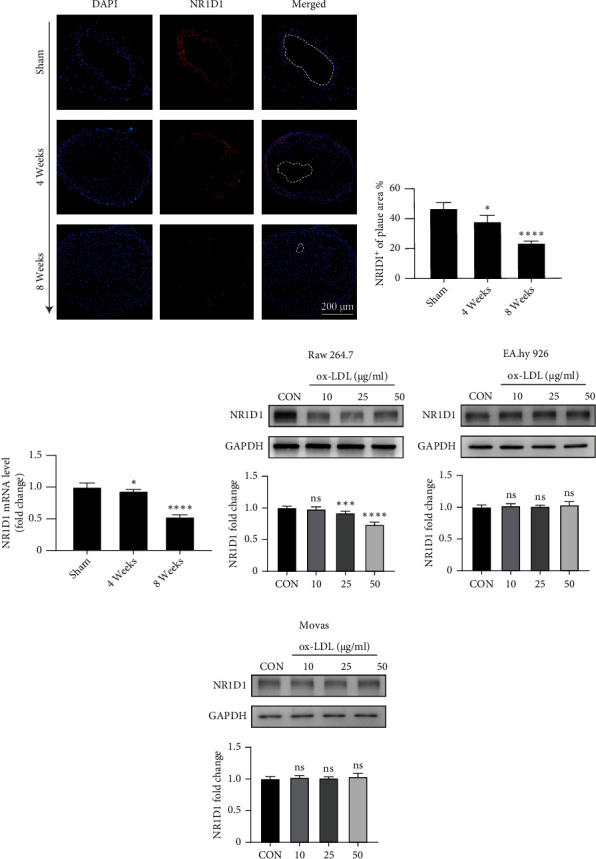
The expression change of NR1D1 during vulnerable plaque formation. (a) The representative immunofluorescence staining of NR1D1 in the mouse carotid artery at different time points after surgery. (b) The quantification of fluorescence intensity of NR1D1 in mouse carotid artery (*n* = 5 per time point). (c) The mRNA expression of NR1D1 in mouse carotid artery at different time points after surgery (*n* = 6 per time point). ^∗^*P* < 0.05 or ^∗∗∗∗^*P* < 0.0001 versus control. (d–f) Western blotting of Raw 264.7, EA.hy 926, and Movas after treatment of ox-LDL for 24 h and quantitative analysis of NR1D1 expression are shown as histograms (*n* = 5 per group). ^∗^*P* < 0.05, ^∗∗∗^*P* < 0.001, or ^∗∗∗∗^*P* < 0.0001 versus control.

To explore whether NR1D1 is involved in the pathological progression of vulnerable plaque, we first investigated the biological roles of NR1D1 in rupture-prone vulnerable carotid plaques. Both immunofluorescence staining of plaque and tissue RT-q-PCR showed that NR1D1 expression levels were significantly reduced in vulnerable carotid plaques (Figures 1(a)–1(c)), suggesting that NR1D1 may exert protective effects in the vasculature. We further explored the expression change of NR1D1 in macrophages, endothelial cells, and vascular smooth muscle cells after ox-LDL administration. NR1D1 expression was not changed in endothelial cells and vascular smooth muscle cells, while it was found to be significantly downregulated after ox-LDL stimulation in macrophages (Figures 1(d)–1(f)). The altered NR1D1 expression in rupture-prone vulnerable carotid plaques implied a potential protective role of NR1D1 in vulnerable plaque progression.

### 3.2. NR1D1 Deficiency Augments Plaque Vulnerability/Rupture

**Figure 2:**
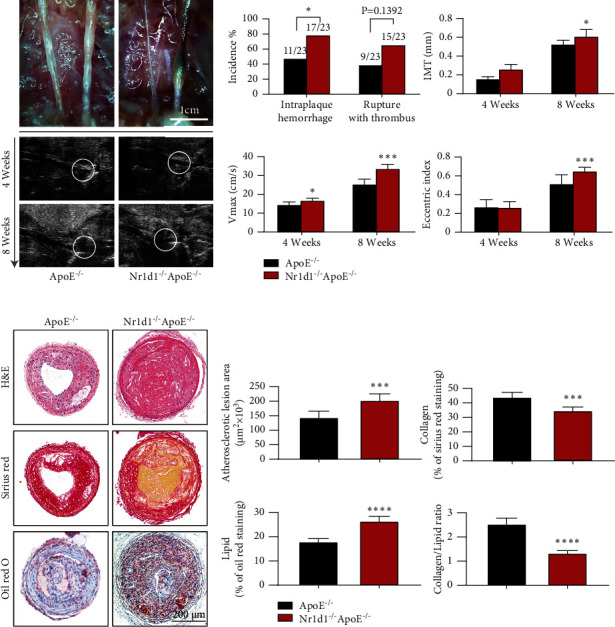
NR1D1 deficiency augments plaque vulnerability/rupture. (a) The representative photographs of the left common carotid arterial segments at 8 weeks after surgery. The arrowhead indicates spontaneous rupture with thrombus and long axis views of the left common carotid arterial segments by microultrasound at 4 weeks and 8 weeks after surgery (*n* = 7 per group); the white circle indicates the vascular lumen. (b) The incidence of intraplaque hemorrhage and rupture thrombus (*n* = 23 per group); the quantification of ultrasound-measured plaque IMT, EI, and *V*_max_ in the ApoE^−/−^ and NR1D1^−/−^ApoE^−/−^ mice (*n* = 7 per group). (c) The representative carotid artery cross-sections were stained with H&E, sirius red, and oil red O. (d) The quantification of the atherosclerotic lesion area and plaque collagen and lipid content. Plaque stability was assessed by the collagen/lipid ratio (*n* = 6–8 per group). ^∗^*P* < 0.05, ^∗∗∗^*P* < 0.001, or ^∗∗∗∗^*P* < 0.0001 versus control.

To determine the detrimental influence of NR1D1 downregulation during vulnerable plaque progression, the mouse model of rupture-prone vulnerable carotid plaques was established (Figure 2(a)) in both ApoE^−/−^ mice and NR1D1^−/−^ApoE^−/−^ mice. We detected a greater carotid plaque burden (Figure 2(a)) in NR1D1^−/−^ApoE^−/−^ mice than in ApoE^−/−^ mice. Moreover, functional analysis with microultrasound imaging showed significantly greater carotid IMT, *V*_max_, and EI in NR1D1^−/−^ApoE^−/−^ mice (Figure 2(b)). Importantly, compared to ApoE^−/−^ mice, NR1D1^−/−^ApoE^−/−^ mice exhibited a higher incidence of intraplaque hemorrhage (78.26% vs. 47.82%, *P* = 0.0325) and spontaneous plaque rupture with thrombus (65.21% vs. 39.13%, *P* = 0.1392) (Figure 2(b)). Apart from that, the greater atherosclerotic area, increased lipid content, and lower collagen/lipid ratio were observed in the carotid plaques of NR1D1^−/−^ApoE^−/−^ mice (Figures 2(c) and 2(d)) compared to ApoE^−/−^ mice. To explore the role of NR1D1 in stabilizing vulnerable plaques, blood pressure, total cholesterol, triglycerides, and body weight were measured and NR1D1 deficiency exerted no significant effects on these parameters (Figure S2). These data suggested that NR1D1 deficiency augmented plaque vulnerability/rupture.

### 3.3. NR1D1 Deficiency Aggravates Macrophage Infiltration and Inflammation in the Vasculature

**Figure 3:**
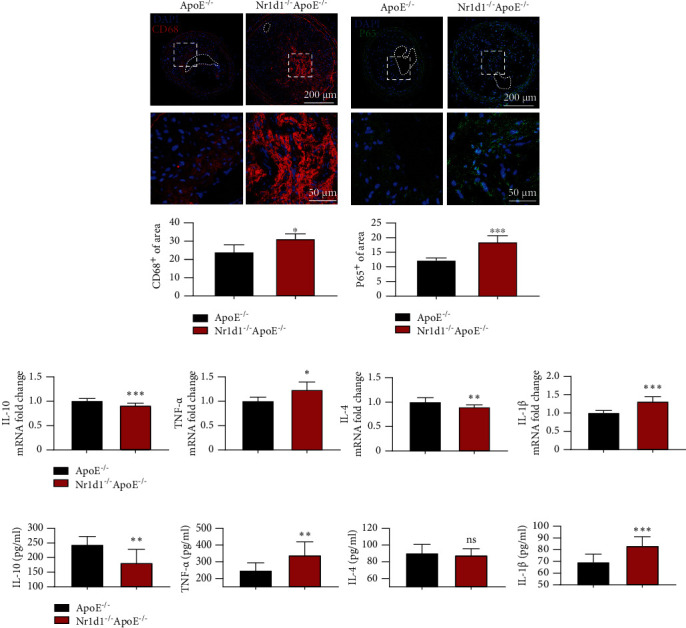
NR1D1 deficiency aggravates macrophage infiltration and inflammation in vasculature. (a) The representative photographs of carotid artery sections that were immunostained with macrophage-specific marker CD68 and the quantification of CD68 in carotid lesions. (b) The representative photographs of carotid artery sections that were immunostained with P65 and the quantification of P65 in carotid lesions. (c) The plasma levels of IL-1*β*, IL-6, IL-4, and IL-10 (*n* = 6 per group). (d) The mRNA expression of proinflammatory cytokines (IL-1*β*, IL-6, MCP-1, and TNF-*α*) and anti-inflammatory cytokines (IL-4 and IL-10) in the mouse carotid artery (*n* = 10 per group). ^∗^*P* < 0.05, ^∗∗^*P* < 0.01, or ^∗∗∗^*P* < 0.001 versus control.

It is reported that NR1D1 could exert anti-inflammatory effects in various diseases [23–25]. We further determined the effects of NR1D1 deficiency on inflammation and macrophage infiltration in the vasculature. Immunofluorescence staining of CD68 showed that macrophage infiltration occurred in vulnerable plaques, which was dramatically augmented by NR1D1 deficiency (Figure 3(a)). Moreover, immunofluorescence staining of plaque revealed that NR1D1 deficiency significantly upregulated NF-*κ*B P65 expression (Figure 3(b)), which indicated an aggravated inflammation in the vasculature. Consistent with increased macrophage infiltration, tissue RT-q-PCR indicated higher levels of proinflammatory cytokines (IL-1*β*, IL-6, MCP-1, and TNF-*α*) and significantly reduced concentrations of anti-inflammatory cytokines (IL-4 and IL-10) in NR1D1^−/−^ApoE^−/−^ mice (Figure 3(c)). The levels of inflammatory factors including IL-1*β* and TNF-*α*in serum samples were significantly upregulated while the levels of anti-inflammatory factors IL-4 and IL-10 were reduced in NR1D1^−/−^ApoE^−/−^ mice (Figure 3(d)). These data suggested that NR1D1 deficiency significantly aggravated the macrophage infiltration and inflammation in carotid artery lesions.

### 3.4. NR1D1 Deficiency Aggravates Oxidative Stress in the Vasculature

**Figure 4:**
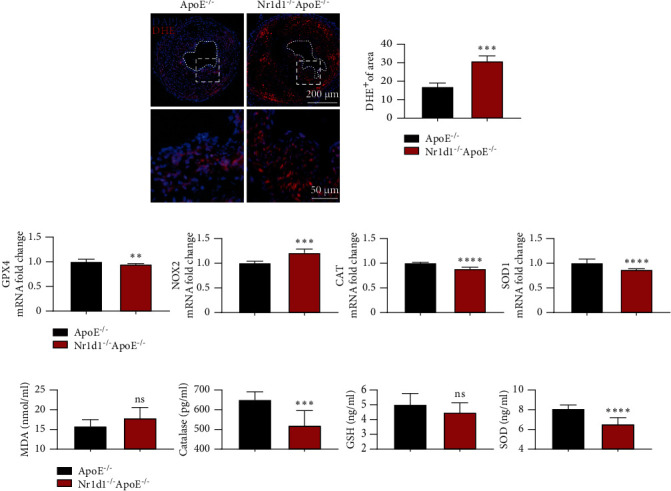
NR1D1 deficiency aggravates oxidative stress in vasculature. (a) The representative dihydroethidium (DHE) staining of carotid artery sections; the quantification of DHE staining intensity in carotid lesions. (b) The plasma levels of MDA, GSH, CAT, and SOD (*n* = 10 per group). (c) The mRNA expression of CAT, SOD1, GPX4, and NOX2 in mouse carotid artery (*n* = 6 per group). ^∗∗^*P* < 0.01, ^∗∗∗^*P* < 0.001, or ^∗∗∗∗^*P* < 0.0001 versus control.

We further evaluated the roles of NR1D1 in regulating oxidative stress in vulnerable plaques. DHE staining indicated that NR1D1 deficiency significantly increased generation of ROS in vulnerable plaques (Figure 4(a)). We further assessed the effects of NR1D1 deficiency on genes related to oxidative stress. The results of tissue RT-q-PCR revealed that several antioxidative genes including CAT, SOD1, and GPX4 were significantly downregulated while NOX2 was upregulated in NR1D1^−/−^ApoE^−/−^ mice (Figure 4(b)). Consistent with that, the levels of oxidative stress-related indicators in serum samples were also significantly increased in NR1D1^−/−^ApoE^−/−^ mice (Figure 4(c)). Collectively, these data suggested that NR1D1 deficiency greatly increased oxidative stress generation in carotid artery lesions.

### 3.5. NR1D1 Deficiency Induces Macrophage Pyroptosis and Vulnerable Plaque Rupture

**Figure 5:**
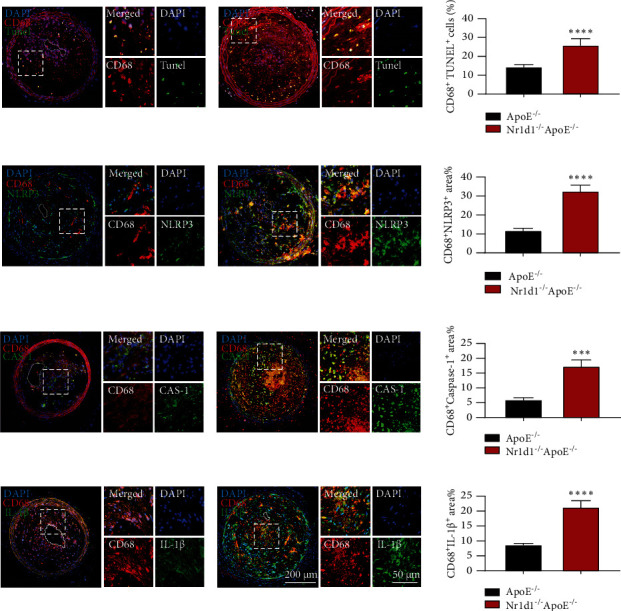
NR1D1 deficiency induces macrophage pyroptosis and vulnerable plaque progression. (a) Carotid atherosclerotic lesions of ApoE^−/−^ and NR1D1^−/−^ApoE^−/−^ mice were probed with specific antibodies against the macrophage marker CD68 and costained with TUNEL staining and the quantification of CD68^+^TUNEL^+^ cells in carotid lesions (*n* = 6 per group). (b) Carotid lesions were stained for NLRP3 and the quantification of NLRP3 in carotid lesions (*n* = 6 per group). (c) Carotid lesions were stained for caspase-1 and the quantification of caspase-1 in carotid lesions (*n* = 6 per group). (d) Carotid lesions were stained for IL-1*β* and the quantification of IL-1*β* in carotid lesions (*n* = 6 per group). ^∗∗∗^*P* < 0.001 or ^∗∗∗∗^*P* < 0.0001 versus control.

Macrophage pyroptosis contributes to plaque vulnerability and promotes plaque rupture [5]. NR1D1 is known to regulate genes involved in pyroptosis [22]. Thus, we further determined the effects of NR1D1 deficiency on macrophage pyroptosis. Interestingly, we observed a significant increase of TUNEL^+^ macrophages in carotid plaque lesions in NR1D1^−/−^ApoE^−/−^ mice (Figure 5(a)). Double immunofluorescence staining indicated that macrophages in carotid plaque lesions in NR1D1^−/−^ApoE^−/−^ mice exhibited increased expression of pyroptosis-related genes such as NLRP3, IL-1*β*, and caspase-1 (Figures 5(b)–5(d)) compared to ApoE^−/−^ mice. Taken together, our results suggested that NR1D1 deficiency activated NLRP3 inflammasome, upregulated IL-1*β* and caspase-1, and thus induced macrophage pyroptosis and rupture-prone vulnerable plaques.

### 3.6. SR9009 Treatment Mitigates ox-LDL-Induced Inflammation, Oxidative Stress, and BMDM Pyroptosis

**Figure 6:**
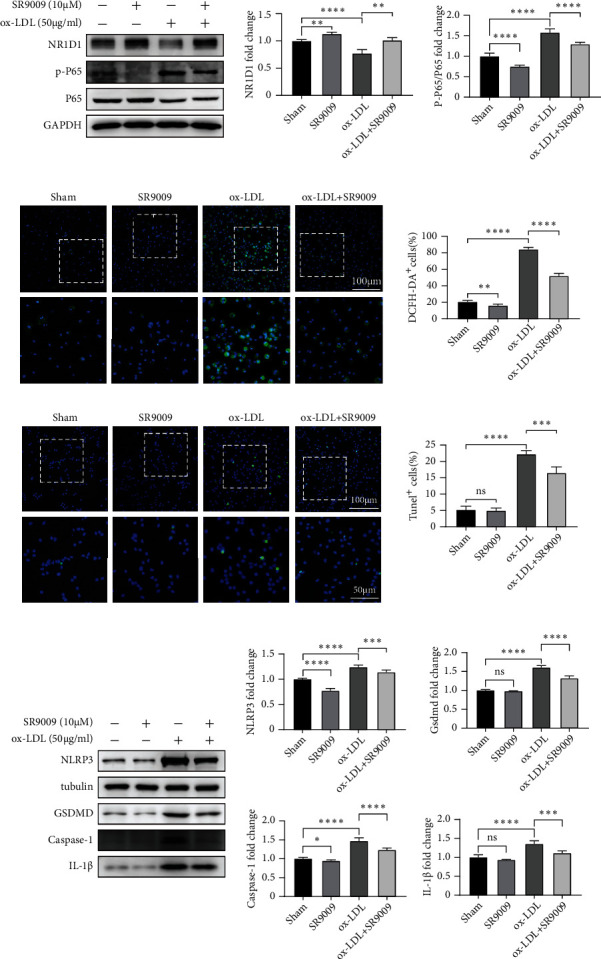
SR9009 treatment mitigates ox-LDL-induced inflammation, oxidative stress, and BMDM pyroptosis. (a) Western blotting of BMDMs after treatment of SR9009 and ox-LDL for 24 h and quantitative analysis of NR1D1 expression and relative densities of phosphorylated P65 compared to total P65 are shown as histograms (*n* = 5 per group). (b) BMDMs were cotreated with SR9009 and ox-LDL for 1 h. ROS generation was measured by DCFH-DA (green); the numbers of DCFH-DA^+^ cells were measured and quantitated. (c) Representative images of TUNEL staining of BMDMs. The number of TUNEL^+^ cells was measured and quantitated. (d) Western blotting of BMDMs after treatment of SR9009 and ox-LDL for 24 h and quantitative analysis of NLRP3, GSDMD, caspase-1, and IL-1*β* expression are shown as histograms (*n* = 5 per group). ^∗^*P* < 0.05, ^∗∗^*P* < 0.01, ^∗∗∗^*P* < 0.001, or ^∗∗∗∗^*P* < 0.0001 versus control.

To explore the potential protective effects of NR1D1 in stabilizing rupture-prone plaques, we first determined the effects of SR9009 (a specific NR1D1 agonist) treatment on ox-LDL-stimulated BMDMs. Consistent with the *in vivo* study, ox-LDL stimulation decreased the expression of NR1D1 in BMDMs (Figure 6(a)). Meanwhile, Western blotting showed that SR9009 decreased the phosphorylation of NF-*κ*B P65 (Figure 6(a)), which indicated an anti-inflammatory effect of SR9009. DCFH-DA assay indicated that SR9009 treatment reduced the ROS generation (Figure 6(b)).

The NLRP3 inflammasome has been identified as a critical regulatory factor in macrophage pyroptosis [26]. It has been demonstrated that NR1D1 regulates the expression of NLRP3 through manipulating transcription. Thus, we further investigated the effects of SR9009 on inflammasome priming. Consistent with the *in vivo* study, SR9009 treatment significantly reduced TUNEL^+^ BMDMs (Figure 6(c)). Moreover, Western blotting showed that SR9009 treatment significantly inhibited ox-LDL-induced pyroptosis-related gene expression (NLRP3, GSDMD, IL-1*β*, and caspase-1) (Figure 6(d)). These observations indicated that upregulation of NR1D1 by SR9009 inhibited macrophage pyroptosis and ROS generation through the NLRP3 inflammasome pathway.

### 3.7. SR9009 Treatment Improves Stability of Rupture-Prone Vulnerable Plaques

**Figure 7:**
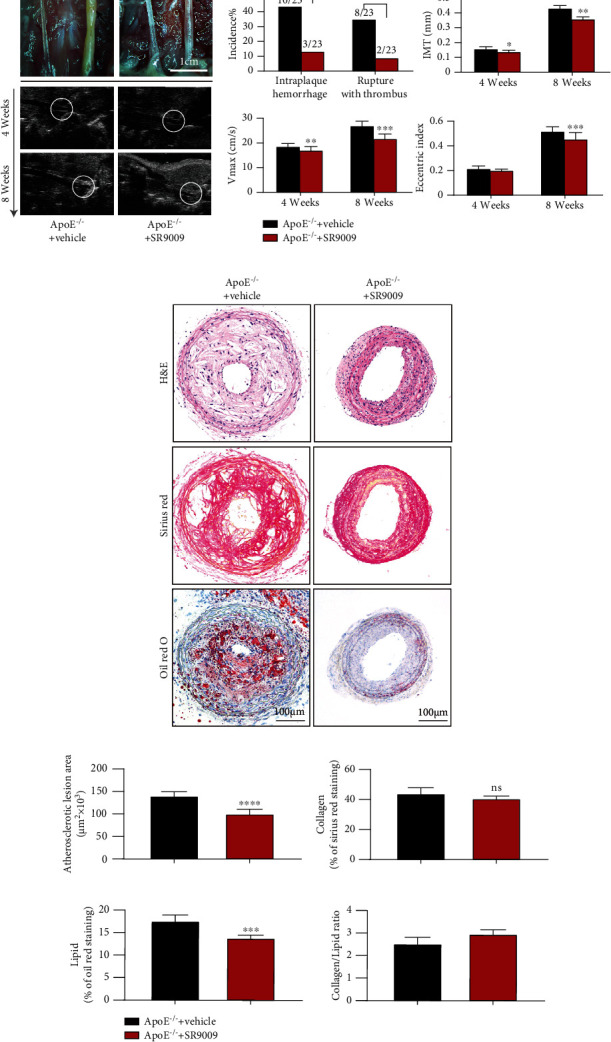
SR9009 treatment improves the stability of rupture-prone vulnerable plaques. (a) The representative photographs of the left common carotid arterial segments at 8 weeks after surgery. The arrowhead indicates spontaneous rupture with thrombus and long axis views of the left common carotid arterial segments by microultrasound at 4 weeks and 8 weeks after surgery (*n* = 6 per group); the white circle indicates the vascular lumen. (b) The incidence of intraplaque hemorrhage and rupture thrombus (*n* = 23 per group); the quantification of ultrasound-measured plaque IMT, EI, and *V*_max_ in the vehicle and SR9009-treated ApoE^−/−^ mice (*n* = 6 per group). (c) The representative carotid artery cross-sections were stained with H&E, sirius red, and oil red O. (d) The quantification of the atherosclerotic lesion area and plaque collagen and lipid content. Plaque stability was assessed by the collagen/lipid ratio (*n* = 6–8 per group). ^∗^*P* < 0.05, ^∗∗^*P* < 0.01, or ^∗∗∗^*P* < 0.001 versus control.

We further determined the protective effects of NR1D1 *in vivo*. SR9009 treatment significantly reduced the incidence of intraplaque hemorrhage (13.04% vs. 43.48%, *P* = 0.0472) and spontaneous plaque rupture with thrombus (8.70% vs. 37.78%, *P* = 0.0706) and thus improved the stability of rupture-prone vulnerable plaque (Figure 7(a)). Moreover, functional analysis with microultrasound imaging verified the protective effects of SR9009. Decreased IMT, *V*_max_, and EI were observed in SR9009-treated mice (Figure 7(b)). The atherosclerotic area, lipid content, and collagen/lipid ratio were also significantly decreased in SR9009-treated mice (Figures 7(c) and 7(d)). Consistent with the *in vitro*study, the levels of inflammatory factors and oxidative stress-related indicators in serum samples were significantly decreased in the SR9009-treated group (Figure S1). These data suggested that SR9009 treatment stabilized rupture-prone vulnerable plaques.

## 4. Discussion

In the present study, we first investigated the role of the nuclear receptor NR1D1 in the vulnerable plaques and demonstrated the protective effects of NR1D1 in the vasculature. By establishing a rupture-prone vulnerable plaque model, we demonstrated that NR1D1 deficiency significantly increased the incidence of intraplaque hemorrhage and spontaneous plaque rupture with the thrombus. Inflammation and oxidative stress were aggravated in NR1D1^−/−^ApoE^−/−^ mice; thus, macrophage pyroptosis was induced. A further study in BMDMs identified the role of NR1D1 in manipulating macrophage pyroptosis by regulating NLRP3 inflammasome. Taken together, our study provided new insights into the significant roles of NR1D1 in the vasculature and suggested that NR1D1 was a unique endogenous protective nuclear receptor against rupture-prone vulnerable plaques by regulating inflammatory response, ROS generation, and macrophage pyroptosis.

Nuclear receptors (NRs) are widely involved in various essential biological functions such as cell growth and death, embryonic development, cellular metabolism, immune response, and inflammation [27, 28]. Mounting evidences suggest that NRs play a pivotal role in maintaining cardiovascular homeostasis, while deficiency of NRs contributes to a wide array of diseases such as atherosclerosis, abdominal aortic aneurysm, and ischemic cardiac injury [18, 29, 30]. NR1D1, a unique member of the nuclear receptor super family, is well known for regulating the circadian rhythm, immune response, and metabolism [31, 32]. Numerous studies have proved that NR1D1 exerted a protective effect by regulating energy metabolism and inhibiting inflammation [33, 34]. Ma et al. found that LDLR^−/−^ mice receiving NR1D1 knockdown bone marrow transplantation developed greater atherosclerotic plaques [35]. Sitaula et al. found that the administration of SR9009 suppressed the progression of atherosclerosis [19]. They both identified that NR1D1 regulated the polarization of macrophages and inhibited inflammation in the vasculature during the atherosclerosis progression. These studies focused on the role of NR1D1 in the progression of atherosclerotic plaque in a mouse model induced with high-fat diet. The majority of cardiovascular events are caused by rupture of vulnerable atherosclerotic plaques [36–38]. At present, there is a general understanding of the initiation and progression of atherosclerosis but the process of the transition from stable plaque to vulnerable plaque remains to be elucidated [2, 39]. Although progress have been made in treating atherosclerosis of the early stage, the effective strategies targeting vulnerable plaques are still lacking [40]. It is of great importance to understand the mechanisms of plaque vulnerability and rupture and to explore the potential drug to stabilize rupture-prone vulnerable plaques. We established the particular rupture-prone vulnerable plaque model to explore the underlying mechanism of plaque vulnerability/rupture. Consistent with former studies, we found that NR1D1 exerted a protective role in the vasculature. Furthermore, we first identified the deficiency of NR1D1-augmented atherosclerotic plaque vulnerability with higher incidence of intraplaque hemorrhage and plaque rupture with thrombus. Targeting NR1D1 by SR9009 significantly prevented the atherosclerotic plaque vulnerability and rupture. In general, our results extended upon the established vasoprotective roles of NR1D1 during atherogenesis and identified SR9009 as a novel drug in stabilizing the rupture-prone vulnerable plaques.

Macrophages play important roles in all stages of atherosclerosis, from the initiation of lesions and lesion expansion, to necrosis leading to rupture and multiple complications [41, 42]. The status of macrophages rather than absolute infiltration number contributes to the inflammation and oxidative stress in the vasculature, leading to plaque vulnerability and rupture [43, 44]. Within the plaques, macrophage pyroptosis induces robust inflammatory response, releases proinflammatory cytokines such as IL-1*β* and IL-18, and thus initiates a deteriorating process causing increased inflammation and oxidative stress [45–47]. Together, these events promote the formation of a necrotic core and thus the vulnerability of the rupture-prone plaques [48]. In addition to macrophages, endothelial cells, smooth muscle cells, etc. also contribute to the progression of atherosclerotic plaques [2]. Whereas macrophage is now considered to play an essential role in the initiation, progression, and especially the final steps of atherosclerosis, namely, plaque vulnerability and eventually plaque rupture [49, 50]. Besides, in vitro experiments showed that ox-LDL stimulation did not alter the expression level of NR1D1 in smooth muscle cells and endothelial cell, while the expression level of NR1D1 in macrophages was significantly downregulated, indicating that NR1D1 mainly manipulates the plaque vulnerability via regulating macrophages.

Inflammasomes including the nucleotide-binding oligomerization domain- (NOD-) like receptor (NLR) family (NLRP3, NLRP1, NLRC4, NLRP9, and NLRP6) and PYHIN protein families (absent in melanoma 2 (AIM2)) detect DAMPs and trigger the canonical pyroptosis signaling pathway [51–53]. Specifically, it has been verified that the NLRP3-caspase-1 signaling pathway is vital in mediating macrophage pyroptosis [54, 55]. DAMPs including inflammatory cytokines and cholesterol crystals activate the NLRP3 inflammasome to bind to adaptor protein apoptosis-associated speck-like protein containing CARD domain (ASC), which recruits procaspase-1. The recruited procaspase-1 is catalytically activated to generate caspase-1 [56, 57]. Active caspase-1 is responsible for processing and maturing IL-1*β*/18 and cleaving the GSDMD to induce macrophage pyroptosis [58]. Indeed, recent studies demonstrated that NR1D1 could regulate the transcription of NLRP3 thus mediating the pyroptosis signaling pathway [20, 22]. We observed that the plaque vulnerability was significantly increased in NR1D1^−/−^ApoE^−/−^ mice, accompanied with increased macrophage pyroptosis in the vasculature. Activation of NR1D1 by SR9009 significantly inhibited macrophage pyroptosis and prevented plaque vulnerability/rupture. In general, our results demonstrated that NR1D1 might manipulate macrophage pyroptosis in a NLRP3-caspase-1-dependent manner within the vulnerable plaques. Recent developments of NR1D1 pharmacology suggest that this nuclear receptor is a druggable target and that ligands targeting NR1D1 may be useful in the treatment of several diseases [12]. Various studies suggest that SR9009, the most common NR1D1 agonist, significantly inhibits inflammation and regulates metabolism and immune response via upregulating NR1D1 [59–61]. Moreover, our results broaden the application fields of SR9009, provide new perspectives for the treatment of vulnerable plaques, and may promote the clinical application of SR9009.

In summary, NR1D1 deficiency aggravated inflammation and oxidative stress in the vasculature and thus induced macrophage pyroptosis and rupture-prone vulnerable plaques. Targeting NR1D1 by SR9009 may represent a novel protective strategy for stabilizing rupture-prone vulnerable plaques.

## Data Availability

Data are available on request.

## Supplementary Materials

Supplementary Materials Table S1: primer sequences used in real-time PCR. Figure S1: SR9009 treatment mitigates the inflammation and oxidative stress. Figure S2: blood pressure and metabolic parameters of ApoE^−/−^ mice and NR1D1^−/−^ApoE^−/−^ mice at the end of the experiments.
